# Melatonin affects red deer spermatozoa motility and physiology in capacitating and non‐capacitating conditions

**DOI:** 10.1111/rda.14137

**Published:** 2022-05-04

**Authors:** Estela Fernández‐Alegre, Estíbaliz Lacalle, Cristina Soriano‐Úbeda, Juan Carlos Domínguez, Adriana Casao, Felipe Martínez‐Pastor

**Affiliations:** ^1^ Bianor Biotech SL León Spain; ^2^ Institute for Animal Health and Cattle Development (INDEGSAL) and IMAPOR Research Group Universidad de León León Spain; ^3^ Department of Molecular Biology (Cell Biology) Universidad de León León Spain; ^4^ Department of Animal Medicine, Surgery and Anatomy (Animal Medicine and Surgery) University of León León Spain; ^5^ Department of Biochemistry and Molecular and Cell Biology Institute of Environmental Sciences of Aragón School of Veterinary Medicine University of Zaragoza Zaragoza Spain

**Keywords:** melatonin, red deer, sperm capacitation, sperm physiology, spermatozoon

## Abstract

Melatonin affects sperm physiology, possibly through membrane receptors. Effects were tested at low concentrations (1 pM, 100 pM, 10 nM and 1 µM) in red deer epididymal spermatozoa as a model for high‐seasonality species. Samples were incubated with melatonin as uncapacitated or capacitating conditions (heparin) and evaluated for motility and physiology (flow cytometry). Most effects occurred at low concentrations (nM‐pM), mainly protecting from apoptosis and maintaining acrosomal integrity, suggesting a role for membrane receptors rather than a direct antioxidant effect. Intracellular calcium was not affected, differing from other studies and perhaps because of the epididymal origin. This study supports the relevance of melatonin on sperm physiology and could contribute to the application of reproductive technologies in wild ruminants.

## 1 INTRODUCTION

Melatonin is a widespread and pleiotropic molecule (Hardeland et al., 2011). Widely studied as a hormone regulating the circadian cycle and seasonality, evidence has mounted on its paracrine activity in reproductive organs (Gonzalez‐Arto et al., 2016; Martínez‐Marcos et al., 2019) and its membrane receptors MT_1_ and MT_2_ are present in spermatozoa (González‐Arto et al., 2016, 2017). Melatonin affects sperm survival, capacitation and chemotaxis (Casao et al., 2012; Cebrián‐Pérez et al., 2014; Espino et al., 2011). This topic has been studied in some domestic ruminants (Fernández‐Alegre et al., 2020; González‐Arto et al., 2016), but it could be different for wild ruminants, subjected to pronounced seasonal reproductive rhythms (Martinez‐Pastor et al., 2005). Our objective was to test its role on red deer (*Cervus elaphus*) spermatozoa under non‐capacitating or capacitating conditions, with the hypothesis that it could affect them at concentrations compatible with receptor activation, as reported for bull (Fernández‐Alegre et al., 2020).

## 2 MATERIALS AND METHODS

The experimental design and methods followed our study on bull (Fernández‐Alegre et al., 2020) and are expanded in the supplementary material. Spermatozoa were obtained by cuts from the cauda epididymis of six adult deers (testicles harvested after regulated hunting at Riaño reserve and Doñana National Park, Spain) (Martinez‐Pastor et al., 2006) and extended in TALP‐HEPES at 50 × 10^6^/ml. Tubes were split, one receiving 2 U/ml heparin (capacitated) and split again, adding melatonin at 1 µM, 10 nM, 100 pM, 1 pM and control (vehicle, 0.2% DMSO).

The samples were incubated for 4 h (38°C, 5% CO_2_) and analysed by CASA for motility (ISAS; Proiser, Valencia, Spain) and flow cytometry. For flow cytometry (CyAn ADP, Beckman Coulter), fluorescent probes were: Hoechst 33,342 (H342), 5 µM; YO‐PRO‐1 (YP1), 100 nM; Fluo‐4 (Fluo4), 100 nM; CM‐H_2_DCFDA (CFDA) at 5 µM; merocyanine 540 (M540), 2 µM; propidium iodide (PI), 1 µM; PNA‐AlexaFluor 647 (PNA), 1 µg/ml; MitoTracker deep red (MT), 100 nM; MitoSOX (MSX), 1 µM. Aliquots were added at 10^6^ ml^‐1^ to combinations of H342/Fluo4/M540/PI/PNA, H342/CFDA/PI and H342/YP/MSX/MT and analysed after 15 min in the dark.

The statistical analysis was performed with R using linear mixed‐effects models (treatment and incubation as fixed effects; male as random effect). Results are shown as mean ± SEM.

## 3 RESULTS

Table 1 summarizes the results for melatonin effects on the non‐capacitated and capacitated groups (further details on the Tables S1 and S2). The proportions of motile spermatozoa (total and progressive) increased in 10 nM only in capacitated samples (*p* < 0.01). Some kinematic variables were significantly affected in non‐capacitated samples by nM‐pM, suggesting a stimulation, with few effects in the capacitated group.

**TABLE 1:** Effect of different melatonin concentrations (1 µM, 10 nM, 100 pM and 1 pM; CTL as the vehicle‐only control, 0.2% DMSO) in non‐capacitating and capacitating conditions

	Non‐capacitated					Capacitated				
Parameter	CTL	1 µM	10 nM	100 pM	1 pM	CTL	1 µM	10 nM	100 pM	1 pM
Total motility (%)	68.7 ± 3.8	70.1 ± 3.5	70.9 ± 2.9	69.0 ± 1.6	72.2 ± 4.1	60.0 ± 2.7	63.3 ± 2.4	70.0 ± 2.3**	61.7 ± 3.7	63.7 ± 2.7
Progressive motility (%)	34.5 ± 3.6	39.5 ± 2.7	38.2 ± 2.5	34.0 ± 1.9	35.9 ± 3.3	30.2 ± 2.8	30.1 ± 2	39.4 ± 2.8**	30.6 ± 2.7	30.6 ± 2.2
VAP (µm/s)	91.7 ± 5.0	98.5 ± 2.6	106.2 ± 1.8**	102.3 ± 2.6	100.9 ± 3.8	112.8 ± 2.2	102.3 ± 4.0*	109.6 ± 3.1	104.4 ± 4.0	104.0 ± 3.9
STR (%)	80.1 ± 1.8	82.1 ± 2.2	80.4 ± 1.8	74.8 ± 2.7*	79.1 ± 1.4	73.0 ± 2.6	74.0 ± 2.3	82.9 ± 3.4*	75.9 ± 3.0	73.8 ± 1.9
ALH (µm)	1.9 ± 0.1	1.9 ± 0.1	2.0 ± 0.0	2.0 ± 0.0*	2.0 ± 0.1**	2.3 ± 0.1	2.1 ± 0.0	2.1 ± 0.0	2.2 ± 0.1	2.2 ± 0.1
Viability (%, PI^–^)	78.7 ± 0.6	77.9 ± 0.7	77.1 ± 1.1	78.6 ± 0.9	80.1 ± 0.5	79.2 ± 0.8	77.4 ± 0.8	75 ± 1.4**	75.7 ± 0.7*	76.1 ± 1.3
Viability (%, YO‐PRO−1^–^)	58.3 ± 1.6	60.1 ± 1.9	57.3 ± 2.7	62.4 ± 2.6	58.8 ± 1.2	45.3 ± 1.0	51.1 ± 1.5*	51.8 ± 2.3*	50.3 ± 2.9*	54.1 ± 1.6**
Apoptotic (%, ratio PI^–^)	23.1 ± 1.7	19.3 ± 3.1	21.5 ± 1.4	14.3 ± 3.8***	22.6 ± 1.1	34.4 ± 2.2	29.5 ± 3.8	24.7 ± 2.4**	30.0 ± 3.2	24.8 ± 1.9**
Reacted acrosomes (%)	29.6 ± 0.7	29.7 ± 0.8	31.2 ± 0.6	25.4 ± 1.9*	25.3 ± 2.2**	34.2 ± 2.4	35.1 ± 2.1	34.0 ± 1.2	36.2 ± 1.8	31.0 ± 1.9
Reacted acrosomes (%, ratio PI^–^)	20.0 ± 0.7	19.7 ± 0.5	20.7 ± 0.3	14.6 ± 2.6**	14.9 ± 2.7**	26.5 ± 2.4	26.3 ± 2.3	23.7 ± 1.7	27.4 ± 2.3	23.0 ± 2.3
Capacitated (%, ratio PI^–^)	17.5 ± 2.4	16.2 ± 1.4	16.5 ± 1.9	15.7 ± 1.3	11.1 ± 0.8**	17.1 ± 1.2	15.8 ± 1.1	16.2 ± 1.2	17.6 ± 0.7	15.3 ± 0.9
Active mitochondria (%)	63.7 ± 1.8	64.4 ± 0.9	63.2 ± 2.5	67.7 ± 0.8	69.0 ± 1.5*	55.8 ± 0.9	59.6 ± 1.6*	57.4 ± 1.8	61.9 ± 0.6**	58.8 ± 0.7
[Ca^2+^] (MFI)	6.7 ± 0.1	7.1 ± 0.2	6.8 ± 0.2	6.7 ± 0.2	6.7 ± 0.2	6.5 ± 0.1	6.4 ± 0.1	6.7 ± 0.2	6.8 ± 0.1	6.6 ± 0.1
Cytoplasmic ROS (MFI)	3.9 ± 0.1	4.0 ± 0.2	4.0 ± 0.1	3.9 ± 0.1	3.7 ± 0.1	3.7 ± 0.1	3.8 ± 0.1	3.8 ± 0.1	3.9 ± 0.1	3.8 ± 0.1
Mitochondrial ROS (%, ratio PI^–^)	21.1 ± 1.9	17.1 ± 0.6	19.1 ± 2.2	11.5 ± 2.9**	18.8 ± 1.7	24.1 ± 1.3	22.7 ± 1.8	19.4 ± 0.6	25.2 ± 2.9	17.5 ± 1.9*

Note

Results are shown as mean ± *SEM*. Significant effects on the control in each group are indicated by *p <.05, **p <.01, ***p <.001. Parameters are described in detail in the main text and the supplementary material.

Sperm viability moderately decreased in capacitated (10 nM and 100 pM, *p* < 0.05), but the proportion of non‐apoptotic spermatozoa significantly increased in all cases, with a drop for the apoptotic ratio in 10 nM and 1 pM (and 100 pM in uncapacitated). Acrosomal damage significantly decreased in uncapacitated by pM. Mitochondrial activity increased by 1 pM in uncapacitated and 1 µM and 100 pM in capacitated. [Ca^2+^]_i_ and cytoplasmic ROS were not affected, but mitochondrial ROS were abated by 100 pM in non‐capacitated (*p* < 0.01) and 1 pM in capacitated samples (*p* < 0.05).

## 4 DISCUSSION

Melatonin affected red deer epididymal spermatozoa at the low range of concentrations, consistent with our previous study on bull semen (Fernández‐Alegre et al., 2020) and exerting a role on sperm physiology through its membrane receptors. In these conditions, a direct antioxidant effect seems unlikely. Our results support previous evidence on a functional role for MT_1_ and MT_2_ in spermatozoa (Casao et al., 2012; Fujinoki, 2008; González‐Arto et al., 2016), consistently showing their implication in sperm capacitation modulation keeping motility and preserving viability and oxidation state.

This first study in red deer spermatozoa hints at a protective effect, possibly by inhibiting apoptosis‐related pathways (Casao et al., 2010; Espino et al., 2011). However, we could not detect intracellular calcium changes, contrarily to studies in ejaculated spermatozoa (Fernández‐Alegre et al., 2020; Gimeno‐Martos et al., 2019). Epididymal spermatozoa are quiescent, not having contacted seminal plasma (Martínez et al., 2008), and signalling pathways modifying calcium patterns could be inhibited at this stage (Ernesto et al., 2015; Gibbs et al., 2011).

This study suggests differences between species and possibly epididymal and ejaculated spermatozoa and could be helpful for the conservation and application of wild ruminant post‐mortem samples.

## CONFLICT OF INTEREST

None declared.

## Supporting information

Supplementary Material
