# Supplementary material for: Melatonin affects red deer spermatozoa motility and physiology in capacitating and non‐capacitating conditions
Source: Reprod Domest Anim. 2022 May 4;57(Suppl 5):82–5. doi: 10.1111/rda.14137 (PMC9790586; doi:10.1111/rda.14137)
Supplement: Supplementary file 1 — Supplementary Material [file RDA-57-82-s001.docx]

**Melatonin affects red deer spermatozoa motility and physiology in capacitating and non-capacitating conditions**

Estela Fernández-Alegre, Estíbaliz Lacalle, Cristina Soriano-Úbeda, Juan Carlos Domínguez, Adriana Casao, Felipe Martínez-Pastor

# S1. Reagents, sample collection, and preparation

General reagents were acquired from Sigma-Aldrich (Merck KGaA, Darmstadt, Germany). Fluorescence probes, purchased from ThermoFisher Scientific (Waltham, MA, USA). Other flow cytometry consumables were purchased from Beckman Coulter (Brea, CA, USA).

The composition of TALP-HEPES medium was: 100 mM NaCl, 3.1 mM KCl, 25 mM NaHCO_3_, 21.6 mM Na lactate, 10 mM HEPES, 5 mM glucose, 3 mM CaCl_2_, 1 mM Na pyruvate, 0.3 mM NaH_2_PO_4_, 0.4 mM MgCl_2_, 2 U/ml gentamicin, 0.5% phenol red, and 0.5% BSA; pH 7.3). The melatonin stock was prepared in DMSO at 50 mM, and then serially diluted so that each tube received 0.2% DMSO (like the control).

The sample collection was carried out as described previously (Fernández-Santos et al., 2007; Martinez-Pastor et al., 2006). The testicles and epididymides were left within the scrotal sack after being harvested from carcasses and sent to the lab refrigerated within 24 h. The samples were dissected in a walk-in fridge (5 °C). Genitals were dissected, isolating the epididymides and vasa deferentia. The epididymis was thoroughly cleaned, and the superficial blood vessels of the cauda were punctured, wiping off blood. The clean epididymides caudae were carefully cut with a scalpel, removing the white fluid from the cut tubules and adding to cold TALP-HEPES. Sperm concentration was assessed with a hemocytometer and adjusted to 50×10^6^ ml^‑1^.

# S2. Motility assessment by CASA (Computer-Assisted Sperm Analysis)

Samples were diluted in TALP-HEPES to 20×10^6^ ml^‑1^ and prepared in a Makler chamber (20–µm depth) with a Nikon E600 (Tokyo, Japan) negative phase contrast microscope on a 37 °C stage at ×10. At least 5 fields and 200 motile cells were recorded using a Basler A312fs digital camera (Basler Vision Technologies, Ahrensburg, Germany) at 53 fps. The ISAS software (Proiser, Valencia, Spain) tracked the spermatozoa and produced Total motility (%), Progressive motility as VCL>25 µm/s and STR>80 (%), VCL (curvilinear velocity; µm/s), VSL (straight path velocity; µm/s), VAP (average path velocity according to the average smoothed path; µm/s), LIN (linearity; %), STR (straightness; %), WOB (wobble; %), ALH (amplitude of the lateral displacement of the sperm head; µm), BCF (frequency of the flagellar beat; Hz), DNC (sperm dance; µm^2^/s), DNCm (sperm mean dance; µm). CASA variables are defined by convention as described previously (Verstegen et al., 2002).

# S3. Flow cytometry analysis of sperm physiology

Fluorescence probes were combined to test sperm physiology as used previously (Crespo-Félez et al., 2017; Fernández-Gago et al., 2013; Ledesma et al., 2016): Hoechst 33342 (H342, debris discrimination) at 5 µM; YO-PRO‑1 (YP1, apoptotic changes in the plasmalemma) at 100 nM; Fluo‑4 (intracellular Ca^2+^ concentration assessment) at 100 nM; CM‑H_2_DCFDA (CFDA, cytoplasmic ROS detection) at 5 µM; merocyanine 540 (M540, capacitation-like plasmalemma changes) at 2 µM; propidium iodide (PI, viability) at 1 µM; PNA‑Alexa Fluor 647 (PNA, peanut agglutinin, acrosomal status) at 1 µg/ml; MitoTracker deep red (MT, mitochondrial activity) at 100 nM; MitoSOX (MSX, mitochondrial superoxide production) at 1 µM. The probes were combined in TALP‑HEPES as H342/Fluo‑4/M540/PI/PNA, H342/CFDA/PI, H342/YP/MSX/MT.

Spermatozoa were added to each probe combination at 10^6^ ml^-1^ and incubated for 15 min in the dark and 37 °C. The samples were run through a CyAn ADP flow cytometer (Beckman Coulter). The flow cytometer was suited with three diode lasers (405 nm, 488 nm, 635 nm). For fluorescence detection, in the 405 nm we used filters 450/50 (H342); in the 488 nm line filters 530/40 (YO‑PRO‑1, Fluo­­‑4, H_2_DCFDA), 575/25 (M540) and 613/20 (PI, MSX); and in the 633 nm line filter 665/20 (PNA‑Alexa Fluor 647, MT). The acquisition was controlled with the Summit V4.3.02 software. The gating strategy included selecting the sperm population in FSC-A/FSC-H, FSC/SSC, and H342/SSC cytograms, using an AND gate for leaving out doublets and debris.

Cytometry data were saved as FCS v.3 files and analyzed with the Weasel v. 3.7 software (Frank Battye, Melbourne, Australia). The variables obtained (%) were: Viability as PI^-^; Viability non-apoptotic YP^–^; Acrosomal damage as PNA^+^; Acrosomal damage ratio as PNA^+^ in the PI^–^ population; Apoptosis ratio as YP^+^ in the PI^–^ population; Capacitation ratio as M540^+^ in the PI^–^ population; Mitochondrial activity as MT^+^/YP^–^ spermatozoa; Superoxide production ratio as MSX^+^ in YP^–^ population. Mean fluorescence intensity (MFI) was used instead of % of cells for estimating intracellular Ca^2+^ concentration ([Ca^2+^]_i_) and cytoplasmic ROS presence (Fluo‑4 and CFDA, respectively) after gating out the PI^+^ population.

# S5. Results

Table S1 compares the non-capacitated and capacitated samples within each melatonin treatment. Most significant differences occurred in the flow cytometry parameters, and none was detected for 10 nM.

The data was also analyzed as the ratio of capacitated respect to non-capacitated samples (Table S2). Notably, the lowest concentrations of 100 and 1 pM affected the ratio to the control for viability, acrosomal integrity (100 pM), capacitation (1 pM), and mitochondrial ROS (100 pM).

Table S1. Significance of the differences between non-capacitated and capacitated treatments within each treatment (* P<0.05; ** P<0.01; *** P<0.001). Refer to Table 1 in the article for actual results as mean±SEM.

| Variable | CTL | 1 µM | 10 nM | 100 pM | 1 pM |
| --- | --- | --- | --- | --- | --- |
| Total motility (%) |  |  |  |  |  |
| Progressive motility (%) |  |  |  |  |  |
| VAP (µm/s) | * |  |  |  |  |
| STR (%) |  |  |  |  |  |
| ALH (µm) |  | * |  |  |  |
| Viability (PI^–^) (%) |  |  |  |  | * |
| Viability (YO-PRO-1^–^) (%) | *** | * |  | * | * |
| Apoptotic (ratio PI^–^) (%) | * |  |  | * |  |
| Reacted acrosome (%) |  | * |  | * | ** |
| Reacted acrosome (ratio PI^–^) (%) | * | * |  | * | * |
| Capacitated (ratio PI^–^) (%) |  |  |  |  | * |
| Active mitochondria (%) | ** |  |  | ** | *** |
| [Ca^2+^] (MFI) |  | * |  |  |  |
| Mitochondrial ROS (ratio PI^–^) (%) |  | * |  | * |  |
| Cytoplasmic ROS (mean) (MFI) |  |  |  |  |  |

| Variable | CTL | 1 µM | 10 nM | 100 pM | 1 pM |
| --- | --- | --- | --- | --- | --- |
| Total motility | 87.2±6.5 | 91.7±7.0 | 99.1±5.2 | 87.6±5.7 | 87.7±6.5 |
| Progressive motility | 88.4±9.1 | 71.3±9.4 | 115.1±18.1 | 91.7±9.4 | 78.5±15.1 |
| VAP | 123.3±8.1 | 104.4±5.3* | 102.9±4.9* | 103.4±5.1* | 106.3±7.9 |
| STR | 90.3±4.5 | 89.6±4.3 | 104.4±6.0 | 103.4±6.0 | 92.6±2.6 |
| ALH | 119.2±5.3 | 109.9±3.4 | 104.1±4.1* | 108.4±3.9 | 106.7±1.8* |
| Viability (PI^–^) | 100.6±1.0 | 99.8±2.0 | 97.3±1.1 | 96.2±0.9* | 94.7±1.7** |
| Viability (YO-PRO-1^–^) | 76.9±1.9 | 84.9±2.3 | 89.8±4.1** | 79.8±2.6 | 91.2±3.5** |
| Apoptotic (ratio PI^–^) | 145.7±15.9 | 143.0±12.8 | 109.7±12.8 | 204.3±28.6 | 107.5±14.4 |
| Reacted acrosome | 114.9±3.9 | 120.2±6.4 | 108.1±5.3 | 138.8±7.7** | 122.9±4.8 |
| Reacted acrosome (ratio PI^–^) | 139.8±16.0 | 138.2±5.1 | 119.4±10.7 | 187.1±11.3** | 152.4±7.6 |
| Capacitated (ratio PI^–^) | 113.5±15.0 | 101.1±14.4 | 107.5±6.0 | 118.3±10.1 | 152.6±15.9* |
| Active mitochondria | 87.7±2.5 | 92.2±3.5 | 92.4±5.5 | 91.6±2.6 | 85.4±2.6 |
| [Ca^2+^] | 99.7±3.8 | 90.9±2.8* | 99.7±1.6 | 104.3±3.4 | 99.6±2.3 |
| Mitochondrial ROS (ratio PI^–^) | 115.3±8.4 | 132.5±13.7 | 111.8±13.4 | 221.1±41.5** | 108.6±20.2 |
| Cytoplasmic ROS (mean) | 94.8±3.8 | 96.4±6.0 | 96.3±3.6 | 99.2±2.8 | 103.8±3.5 |

Table S2. Ratios for capacitated/non-capacitated red deer spermatozoa (* P<0.05; ** P<0.01; *** P<0.001). Refer to Table 1 in the article for actual results as mean±SEM.

# S6. References

Crespo-Félez, I., Castañeda-Sampedro, A., Sánchez, D. I., Fernández-Alegre, E., Álvarez-Rodríguez, M., Domínguez, J. C., Morrell, J. M., & Martínez-Pastor, F. (2017). Effect of Single Layer Centrifugation Porcicoll (70%, 80% and 90%) or supplementation with reduced glutathione, seminal plasma and bovine serum albumin on frozen-thawed boar sperm. *Animal Reproduction Science*, *187*, 167–173. https://doi.org/10.1016/j.anireprosci.2017.11.002

Fernández-Gago, R., Domínguez, J. C., & Martínez-Pastor, F. (2013). Seminal plasma applied post-thawing affects boar sperm physiology: A flow cytometry study. *Theriogenology*, *80*(4), 400–410. https://doi.org/10.1016/j.theriogenology.2013.05.003

Fernández-Santos, M. R., Martínez-Pastor, F., García-Macías, V., Esteso, M. C., Soler, A. J., de Paz, P., Anel, L., & Garde, J. J. (2007). Extender osmolality and sugar supplementation exert a complex effect on the cryopreservation of Iberian red deer (*Cervus elaphus hispanicus*) epididymal spermatozoa. *Theriogenology*, *67*(4), 738–753. https://doi.org/10.1016/j.theriogenology.2006.10.005

Ledesma, A., Fernández-Alegre, E., Cano, A., Hozbor, F., Martínez-Pastor, F., & Cesari, A. (2016). Seminal plasma proteins interacting with sperm surface revert capacitation indicators in frozen-thawed ram sperm. *Animal Reproduction Science*, *173*, 35–41. https://doi.org/10.1016/j.anireprosci.2016.08.007

Martínez-Pastor, F., García-Macías, V., Alvarez, M., Chamorro, C., Herráez, P., de Paz, P., & Anel, L. (2006). Comparison of two methods for obtaining spermatozoa from the cauda epididymis of Iberian red deer. *Theriogenology*, *65*(3), 471–485. https://doi.org/10.1016/j.theriogenology.2005.05.045

Verstegen, J., Iguer-Ouada, M., & Onclin, K. (2002). Computer assisted semen analyzers in andrology research and veterinary practice. *Theriogenology*, *57*(1), 149–179.
